# Intermediate Syndrome and Marchiafava-Bignami Syndrome: Double Trouble in Weaning Off

**DOI:** 10.7759/cureus.26694

**Published:** 2022-07-09

**Authors:** Ruchita Kabra, Maharshi Patel, Pratik J Bhansali, Sunil Kumar, Sourya Acharya

**Affiliations:** 1 Department of Medicine, Jawaharlal Nehru Medical College, Wardha, IND; 2 Department of Radiodiagnosis, Datta Meghe Institute of Medical Sciences (Deemed to be University), Wardha, IND; 3 Department of Medicine, Jawaharlal Nehru Medical College/Datta Meghe Institute of Medical Sciences (Deemed to be University), Wardha, IND

**Keywords:** marchiafava-bignami disease, weakness, organophosphorus poisoning, intermediate syndrome, respiratory failure

## Abstract

Intermediate syndrome affects 10-40% of those with severe organophosphorus poisoning, causing delayed weakness in the proximal parts of the body, neck flexors, and breathing muscles. We present the case of organophosphorus poisoning that advanced to intermediate syndrome and subsequently worsened, with imaging later revealing the Marchiafava-Bignami condition, which aggravated the intermediate syndrome.

## Introduction

Intermediate syndrome (IMS) is a cluster of signs and symptoms that arise after the acute cholinergic syndrome has passed. The IMS develops in about 20% of patients post oral exposure to organophosphate pesticides, with no definite link between the organophosphate chemical used and the onset of the syndrome [1]. It usually takes two to four days to establish itself after being exposed to organophosphate (OP) pesticides. Acute organophosphate (OP) self-poisoning has serious neurologic consequences, such as coma, seizures, and muscle paralysis, as well as respiratory failure induced by cholinergic crisis. It's also more likely to be consumed while under the influence of alcohol. Marchiafava-Bignami disease (MBD) is a rare neurological disease characterized by necrosis and demyelination in callosal lesions [2-4]. It is commonly linked to prolonged, severe alcohol intake and malnutrition. We present a case of organophosphorus poisoning that advanced to intermediate syndrome and subsequently worsened, with imaging later revealing the Marchifava-Bignami condition, which aggravated the intermediate syndrome.

## Case presentation

A 24-year-old male patient was admitted with complaints of profuse sweating, vomiting, cough, and sialorrhoea and a history of consumption of monosul-36 containing monocrotophos compound, an organophosphorus compound at his farm. The patient was chronic alcoholic for eight years and consumed alcohol daily. The patient consumed organophosphorus compounds under the influence of alcohol. On admission, general condition was poor, tachypneic with respiratory rate of 20 breaths/min, heart rate of 60 beats/min, regular, blood pressure (BP) was 130/90 mm Hg in right arm supine position, temperature was 36.8°C, oxygen saturation was 90% on room air. Respiratory system examination showed bilateral crackles. He was drowsy with altered sensorium having pupils 1 mm reactive to light, and fasciculations were noticed over thigh. Other systems were normal. He had a history of seizure episodes in the past and memory loss.

He was admitted to the medicine intensive care unit for constant observation and monitoring. As the patient was already drowsy and not maintaining saturation, he was intubated and taken on ventilator. Laboratory investigations done are mentioned in Table 1, of which serum cholinesterase is suggestive of organophosphorus poisoning with severe thiamine and vitamin B12 deficiency and albumin deficiency.

**Table 1:** Investigations of the patient at the time of admission

Laboratory parameters	Values	Reference range
Hemoglobin	11.2 g%	12.5-14.5 g%
White blood cell count	11,700/cu mm	6000-11,000/cu mm
Platelet count	99,000/mcL	1,50,000-4,50,000/mcL
Mean corpuscular volume (MCV)	108.2 fL	80-100 fL
Aspartate transaminase (AST)	215 u/L	<37 u/L
Alanine transaminase (ALT)	92 u/L	<42 u/L
Serum albumin	2.1 g/dL	3.5-5.0 g/dL
Serum bilirubin	4.5 mg/dL	0.2-1.2 mg/dL
Serum urea	32 mg/dL	9-20 mg/dL
Serum creatinine	0.9 mg/dL	0.6-1.2 mg/dL
Serum sodium	144 mmol/L	137-145 mmol/L
Serum potassium	3.8 mmol/L	3.5-5.1 mmol/L
Serum vitamin B12	137 pg/mL	239-930 pg/mL
Serum cholinesterase	0.3 U/mL	5.9-12.2 U/mL

The patient was managed with atropine and injection pralidoxime infusion and other symptomatic management. On regular examination, the patient’s chest was found to be clear and injection atropine infusion was tapered and stopped, and also, the patient was weaned off ventilator and taken on T-piece. After two hours of atropine infusion stoppage, the patient was found drowsy again and not maintaining oxygen saturation on T-piece, so he was again started on volume control mode.

**Figure 1:**
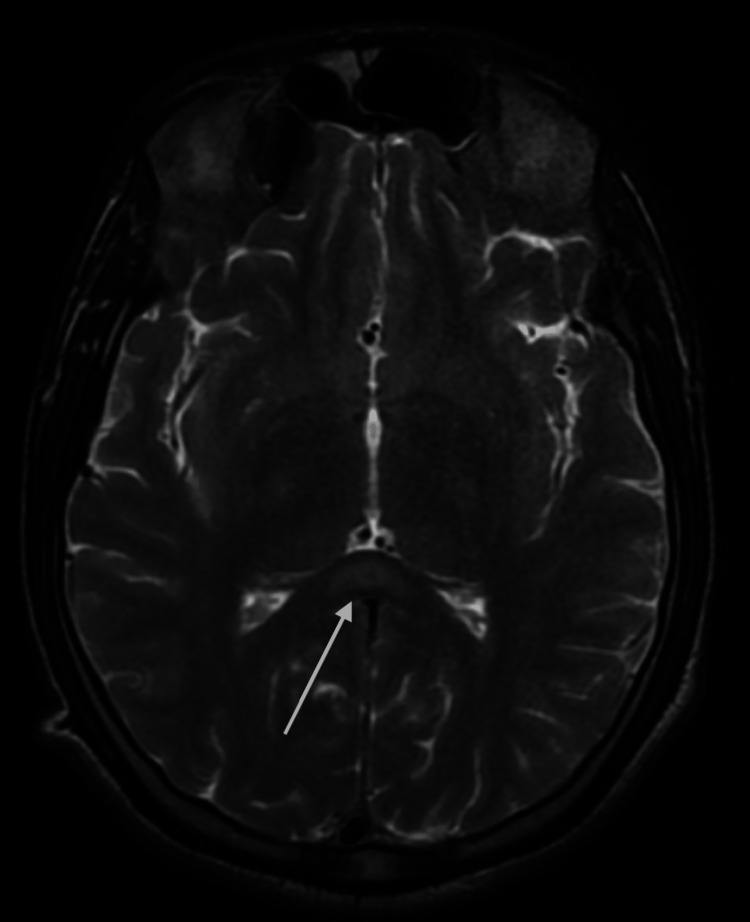
MRI imaging showing hyperintense signal on the T2WI T2WI: T2-weighted

**Figure 2:**
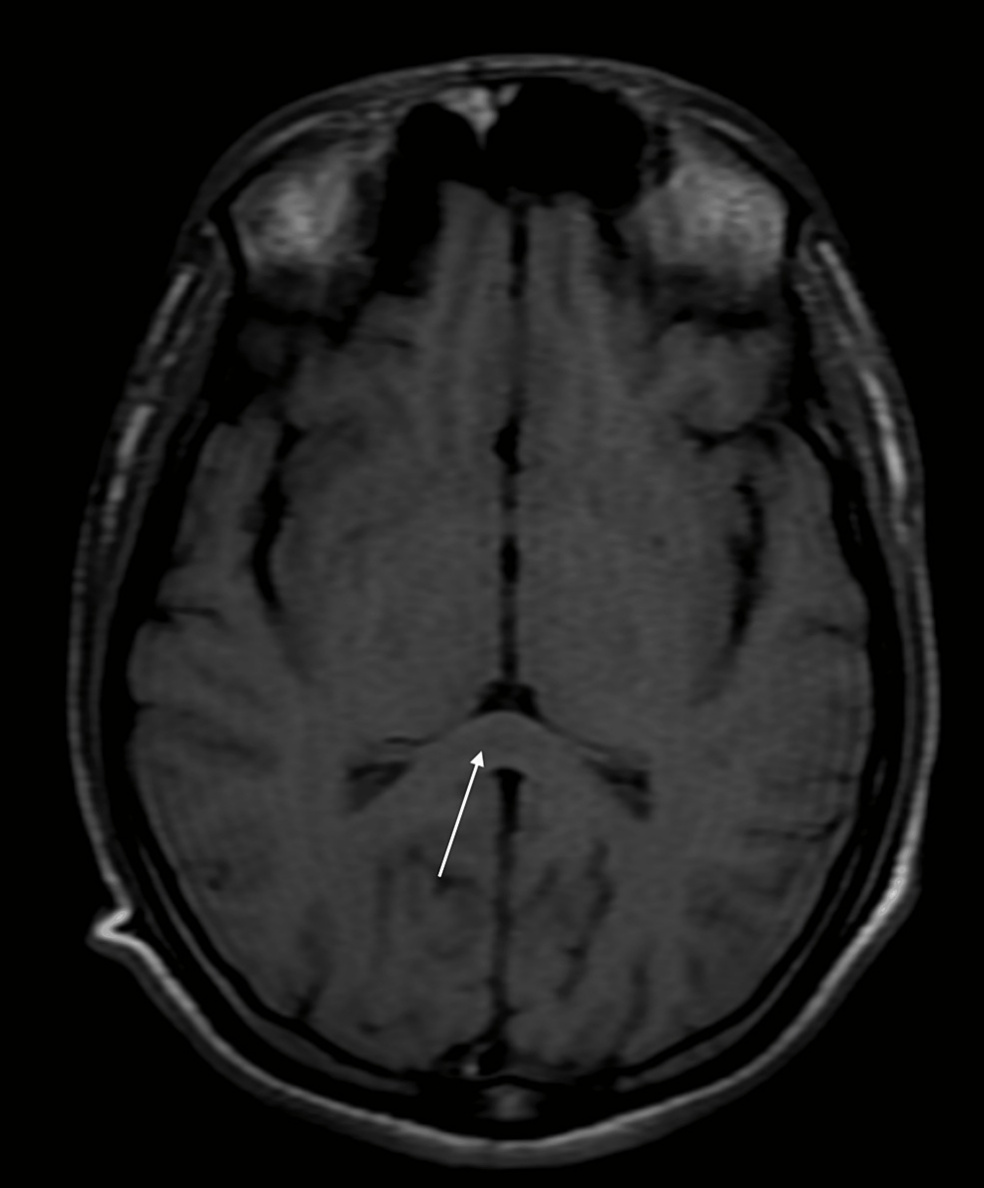
MRI imaging showing hypointense signal on the T1WI T1WI: T1-weighted

Atropine infusion was restarted at 2 mg/h. Multiple attempts were done to wean off ventilator, but they were unsuccessful; this was found to be respiratory muscle weakness along with other proximal muscle weaknesses which is suggestive of intermediate syndrome. MRI brain was planned as the patient was drowsy in spite of fully atropinization and no evidence of any sepsis, which revealed focal lesion in the splenium of corpus callosum including hyperintensity signal on the T2WI (Figure 1) and hypointensities on the T1W1 (Figure 2). On the basis of this finding, Marchiafava-Bignami syndrome (MBD) diagnosis was kept.

On sixth day, tracheostomy was planned due to persistent respiratory muscle weakness. Tracheostomy was done and the patient was taken on continuous positive airway pressure (CPAP) mode and was maintaining oxygen saturation so was shifted to T-piece. As his cholinergic symptoms were gradually improving, antidote therapy of atropine and pralidoxime infusion were stopped. In view of vitamin B12 deficiency, intravenous administration of thiamine was given which relieved the patient of deficient state. Consequently, the patient started showing improvement as he became fully alert and responding to all the commands. Eventually, the patient was weaned off from the ventilator and tracheostomy was closed, and the patient is doing well on follow-up.

## Discussion

Intermediate syndrome occurred after the intense cholinergic difficulty had subsided but before the onset of organophosphorus (OP) poisoning induced peripheral neuropathy. It appears 12-72 hours after organophosphorus poisoning and can last for up to five to six days. This syndrome is characterized by a sudden onset of muscle weakness that includes the respiratory muscles (particularly the diaphragm), as well as a lack of mobility in the neck muscles (inability to elevate the head from the pillow) and proximal limb muscle weakness. Certain cranial nerve palsies, such as those affecting the external ocular, jaw, facial, and palatal muscles, are occasionally detected [5].

Lack of pralidoxime treatments and tissue redistribution could be the culprits, but these theories aren't universally accepted [6]. After the intense cholinergic stage, a second phase of weakness occurs after one to four days and, if left untreated, can lead to fatal breathing problems [7]. If respiratory failure occurs, intubation and mechanical ventilation are required. Breathing difficulty is one of the harmful cholinergic characteristics of organophosphate poisoning. The time spent on ventilatory support ranges from five to seven days. The patient had been on a mechanical ventilator for 10 days and had difficulties weaning off, despite the fact that he was completely alert. By increasing elastic stress of breathing (more work is required to inflate the already overinflated lung) and diminishing diaphragmatic function, worsening dynamic hyperinflation also predisposes to difficulty weaning. Critical illness neuromyopathy, electrolyte abnormalities (hypomagnesemia, hypocalcemia, hypokalemia, hypophosphatemia), medications (steroids-induced myopathy, neuromuscular blocking agents), ventilator-induced diaphragmatic dysfunction (secondary to excessive ventilatory support), hypothyroidism, adrenal insufficiency, and sepsis are some of the possible causes [8]. However, in this example of organophosphorus poisoning, Marchiafava-Bignami disease (MBD) was a probable cause for prolonged weaning from intermediate syndrome.

Marchiafava-Bignami disease (MBD) is a rare neurological disease characterized by necrosis and demyelination in callosal lesions. It is often linked to chronic, heavy alcohol consumption and malnutrition [2-4]. The most common theory for the cause of Marchiafava-Bignami disease is that it is caused by malnutrition or a vitamin B deficiency [8].

Because Marchiafava-Bignami disease is still considered a rare disease with no typical symptoms, it is difficult to diagnose and distinguish it from other disorders. The diagnosis is based on a clinical, pathological, and radiological correlation. In a persistent alcoholic/malnourished patient presenting with neurological problems, a high level of suspicion is justified. Thiamine deficiency and callosal lesions on MRI clinch the diagnosis. Coma, death, or other significant neurocognitive abnormalities may ensue from a delay in appropriate diagnosis. MRI also aids in the prediction of prognosis in diagnosed cases and eliminates other Marchiafava-Bignami disease differential diagnoses.

## Conclusions

Marchiafava-Bignami disease could be a reversible brain condition. Clinicians should raise awareness of the condition and underline that it cannot be overlooked in patients who have never drank, especially those who are malnourished. However, in a patient with organophosphorus poisoning and intermediate syndrome, especially in patients with prolonged drinking, this entity must be considered.
